# Acid-Sphingomyelinase Triggered Fluorescently Labeled Sphingomyelin Containing Liposomes in Tumor Diagnosis after Radiation-Induced Stress

**DOI:** 10.3390/ijms22083864

**Published:** 2021-04-08

**Authors:** Carola Heneweer, Tuula Peñate Medina, Robert Tower, Holger Kalthoff, Richard Kolesnick, Steven Larson, Oula Peñate Medina

**Affiliations:** 1 Department of Diagnostic and Interventional Radiology, Faculty of Medicine, University Hospital Cologne, 50937 Cologne, Germany; germanycarola.heneweer@uk-koeln.de; 2 Department of Radiology and Neuroradiology, University Medical Center Schleswig-Holstein (UKSH), Kiel University, 24118 Kiel, Germany; 3 Section Biomedical Imaging, Molecular Imaging North Competence Center (MOIN CC), Department of Radiology and Neuroradiology, University Medical Center Schleswig-Holstein (UKSH), Kiel University, 24118 Kiel, Germany; tuula.penate@rad.uni-kiel.de (T.P.M.); robert.tower@rad.uni-kiel.de (R.T.); 4 Institut für Experimentelle Tumorforschung (IET), Arnold-Heller-Str. 3, Haus U30, 24105 Kiel, Germany; hkalthoff@email.uni-kiel.de; 5 Laboratory of Signal Transduction, Memorial Sloan-Kettering Cancer Center, New York, NY 10065, USA; r-kolesnick@ski.mskcc.org; 6 Department of Radiology, Sloan Kettering Institute for Cancer Research, New York, NY 10065, USA; lar-sons@mskcc.org

**Keywords:** controlled release, drug delivery, sphingomyelinase, cell stress, quenching, fluorescence, liposome

## Abstract

In liposomal delivery, a big question is how to release the loaded material into the correct place. Here, we will test the targeting and release abilities of our sphingomyelin-consisting liposome. A change in release parameters can be observed when sphingomyelin-containing liposome is treated with sphingomyelinase enzyme. Sphingomyelinase is known to be endogenously released from the different cells in stress situations. We assume the effective enzyme treatment will weaken the liposome making it also leakier. To test the release abilities of the SM-liposome, we developed several fluorescence-based experiments. In in vitro studies, we used molecular quenching to study the sphingomyelinase enzyme-based release from the liposomes. We could show that the enzyme treatment releases loaded fluorescent markers from sphingomyelin-containing liposomes. Moreover, the release correlated with used enzymatic activities. We studied whether the stress-related enzyme expression is increased if the cells are treated with radiation as a stress inducer. It appeared that the radiation caused increased enzymatic activity. We studied our liposomes’ biodistribution in the animal tumor model when the tumor was under radiation stress. Increased targeting of the fluorescent marker loaded to our liposomes could be found on the site of cancer. The liposomal targeting in vivo could be improved by radiation. Based on our studies, we propose sphingomyelin-containing liposomes can be used as a controlled release system sensitive to cell stress.

## 1. Introduction

Successfully targeted drug delivery is among the most crucial pharmaceutics goals. In the targeted approach, the drug or marker molecule is strictly localized to the site or organ of action, offering a pharmacological activity to the needed area. Well accomplished targeting decreases the required drug dose and systemic toxicity and increases treatment efficacy. Often targeting is challenging, while targets are challenging to reach for anatomic reasons, particularly by insufficient blood supply or high drug resistance. Despite recent advances in nanoparticle probe development, the translation of targeted diagnostic particle platforms remains challenging. The most used nanostructures for drug delivery in the clinic include liposomes and micelles [1]. From nanoparticle-based materials currently under evaluation in oncological clinical trials, most are non-targeted drug delivery vehicles using enhanced permeability and retention (EPR) effect to reach tumor sites. What is lacking in clinical use is a well-controlled release system that can unload the drug cargo in the area of interest. Nanoparticles have to be non-toxic and somehow increase the drug effect by improving pharmacokinetics [1]. Liposomal nanoparticles have an advantage while they resemble naturally occurring lipid structures like exosomes. The closest clinically tested activatable candidate is thermally activated liposomes, where external heat is applied on the release site [2]. This approach has problems, while the thermal range between average body temperature, liposome phase transition temperature, and tissue harming temperature is tight. Because novel drug delivery systems are complicated, imaging techniques could bring the extra knowledge needed to evaluate the delivery efficacy. Molecular imaging techniques could elucidate tumor-selective probes and specify the targeted interactions between the microenvironment and the probe. Molecular imaging is also critical to the development of targeted molecular medicine because it could allow early detection of diseases, monitoring drug efficacy, and identifying the disease. Therefore, there is a strong need to develop in vivo nanoprobes for drug targeting, functional imaging, and targeted therapy.

We have developed an environment-sensing liposome, which we wanted to study in vitro and in vivo using mainly optical techniques. The biologically relevant lipid, sphingomyelin (SM), was included in the liposomal membrane. SM is a common lipid within cells typically situated primarily on the cell membrane’s outer surface [3]. Interestingly, katabolic product of SM, ceramide, is a very versatile lipid by its biological functions (reviewed by Stancevic and Kolesnick [4]). The change from SM to ceramide affects biophysically to the membrane making it leakier [5]. Several model membrane studies also show an increase in ceramide levels destabilizing membranes’ bilayer structure [6,7,8,9]. Here, we demonstrate that it is possible to design SM-containing liposomes that release their payload upon contact with the sphingomyelinase (SMase) enzyme.

Interestingly, this enzyme responsible for SM catabolic [10] is also relevant in biological functions [11]. Secreted acid sphingomyelinase is involved in several different stress reactions like under radiation [4,12]. The ASMase is released from lysosomes in vesicles, in stress situations, while it can perform vital cell regulation and membrane repairmen functions on the outer cell membrane [13]. The apoptotic cells transport SMase to the apoptotic cells’ outer leaflet [14], which does not occur in healthy cells. Because acid sphingomyelinase is secreted to the cellular plasma membrane or secreted to the surroundings in stressful situations and disease conditions, we wanted to use this activity to make a delivery vehicle, which will be structurally weakened by sphingomyelinase. The weakening is possible if SM-containing liposome is used and finds its way to the stressed disease site. The enzymatic environment helps the liposome in the release process by acting on liposomal SM. We tested in vitro if sphingomyelinase action on the liposomal membrane can release the loaded fluorescent markers from the SM-liposome. Here, we describe a method for accurate SMase activity detection by using liposomes that can be activated by SMase. The study was done with a liposome loaded with fluorescent marker molecules and verified using liposomes loaded with radioactively labeled BSA. Based on our results here, we can conclude that sphingomyelinase treatment actually increases the release of fluorescent markers from liposomes. We show that SMase secretion will occur in the acute stress caused by irradiation in the HAoEK and PC-3 cells. This phenomenon can be used for site-specific disruption of the SM-containing liposomes while the PC-3 cells and HAoEK cells secrete SMase in a dose-dependent manner after irradiation. Another question we raised was SM-liposomes can be used in tumor targeting. We studied in PC-3-tumor mouse model the biodistribution of the SM-liposomes and could conclude that SM -liposomes can be used in tumor targeting and stress caused by radiation increases the targeting efficacy. We suggest that the site-targeted liposomal delivery can be used for drug delivery in all possible conditions where cell stress induces SMase secretion. In drug delivery, this will create a potential delivery system that will bring the drug available only to the stressed cells with the acute need of the drug. An advantage of the proposed system is the possible amplification of the signal since one enzyme will cleave thousands of SMs into ceramides on the surface of the carrier liposome. As a result, the liposomes rupture and release their payload to the extracellular or intracellular compartment.

## 2. Results

### 2.1. Liposome Preparation 

**Figure 1:**
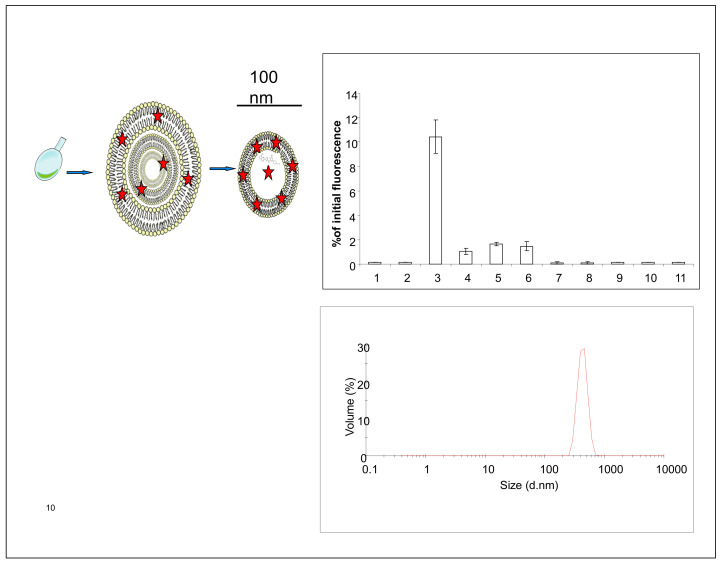
Panel 1 shows a schematic presentation of SM-containing liposomes and their preparation. Liposomes are dried into the round-bottom glass vial (most left image). In a hydration step, multilamellar liposomes are formed (middle image). After extrusion, unilamellar liposomes with a 100 nm diameter are formed (image on the right). Stars represent positions where fluorescent dye or drug can be added depending on their hydrophobic nature. (upper right) shows a typical elution curve after the liposomal purification with size exclusion column chromatography. Fluorescence, here cy5.5, is used to track the liposomes, which come out in the third fraction. (below) presents a typical size distribution of SM liposomes analyzed with dynamic light scattering.

Sphingomyelin containing unilamellar liposomes were prepared as described earlier [15] and shortly presented in Figure 1. The liposomal encapsulation studies revealed (Figure 1) that fluorophores, and I-124 tyrosine labeled bovine serum albumine (BSA) [16] were relatively easy to encapsulate into the liposomes. Overall, the material can be loaded into the lumen of liposomes if the material is water-soluble. If the material is hydrophobic, it can be loaded/attached to the lipid membrane (Figure 1). Prepared SM-liposomes were controlled using dynamic light scattering (DLS) for size measurements. The liposomes had an average hydrodynamic diameter of 100–200 nm (Figure 1 bottom). Different fluorescent tracers (Alexa 680, Cy 5.5, and beacon containing hairpin) were loaded to the liposomes to study the release of the loaded markers from liposomes after the liposomes were treated with sphingomyelinase enzyme in vitro. The study was finalized in vivo and the fluorescent liposomes were traced from tumors.

### 2.2. In Vitro Release Assay

**Figure 2:**
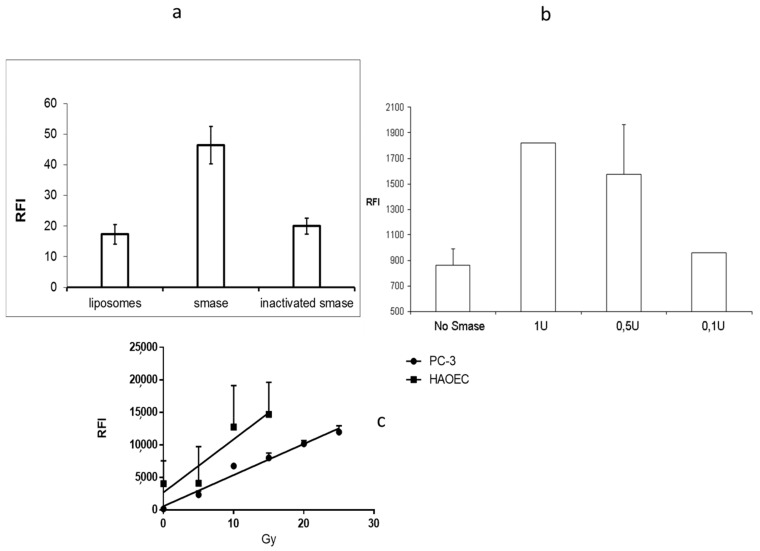
(**a**) Panel 2a presents the fluorescence of Cy 5.5 loaded liposomes 30 min after the sphingomyelinase treatment (middle column) as a control, liposomes were treated similarly but with heat-inactivated sphingomyelinase (column on the right). The column most left presents the liposomal fluorescence before any treatment. (**b**) The graph on panel 2b presents the leakage caused by sphingomyelinase when CY7-Dabcyl hairpin was encapsulated in SM-liposomes. Fluorescence can be seen when hairpin is released from the liposomal lumen and it meets the target DNA in the medium outside the liposomes. Three SMase concentrations (1 U, 0.5 U, and 0.1 U) were used in the study, and non-treated liposomes (far right) were used as a control. The graph in panels (**c**) represents secreted acid sphingomyelinase activity after radiation treatment of HAoEC cells (■) and from PC-3 cells (●). The cells were irradiated with various radiation (5-15 Gy in HAoEC and 5-25 Gy in PC-3) doses. SMase was detected by using the optical SMase activity-kit. The error bars in all graphs are SEM.

The performed release studies are based on molecular quenching [17]. When fluorescent molecules are tightly packed, the fluorescence is partly silenced while some of the electron excitations are hindered. If the molecular packing is opened or the content released, like in the case of liposomes, the release can be seen as an increase in fluorescence. The quenching was analyzed when SM-containing liposomes were treated with sphingomyelinase enzyme. The sphingomyelinase acts on SM cutting the phosphocholine head group forming ceramide. Ceramide is a unique lipid in many ways [4]. It has several biological roles, while it is known to be involved, among other things, in cell growth and apoptosis and viral or bacterial entry processes [18,19,20]. Besides, ceramide has a special kind of three-dimensional structure, which makes ceramide a non-bilayer lipid. When enough ceramide is added into the model membrane, ceramide causes invaginations [8], domain formation, and as shown earlier, the liposomes become leakier [5]. Cy 5.5 is a fluorescent probe, which is quenched when packed in high concentration into liposomes. SM-liposomes loaded with Cy 5.5 were incubated with different amounts of SMase, and the change in fluorescence was analyzed. Figure 2a shows a statistically relevant increase in fluorescence after sphingomyelinase addition to the liposomal solution. The heat-inactivated SMase did not cause a similar effect. The data confirm that quenching of the Cy 5.5 fluorescence inside the liposomes could be overcome if liposomes were treated with active SMase, which ruptures the liposome membrane. This causes the release of dye. Heat inactivated SMase did not affect the fluorescence, indicating that enzymatic activity is needed for the membrane rupture. The quenching assay was verified using another release system: A DNA beacon containing a hairpin loop with a CY7-Dabcyl quenching pair. This loop can be opened with a short DNA fragment that binds to the loop, forces the loop to open, and separates the Cy7 and Dabcyl quencher releasing fluorescent light. Hairpin was encapsulated inside the liposome and the hairpin opening DNA sequence was in the buffer outside of the liposomes. By measuring fluorescence, we could see if the hairpin was released from the liposomal lumen and opened by the DNA sequence laying in the outside buffer.

Three SMase concentrations (1 U, 0.5 U, and 0.1 U) were used to release the hairpin to the outside media, where the target DNA was located (Figure 2b). The results show a dose-responsive increase of fluorescence after the SMase treatment on SM-containing liposomes. Both release assays described reveal that sphingomyelinase outside the liposome can make the liposome leakier and cause the release of loaded materials to the liposomal surroundings.

### 2.3. In Vitro Acid Sphingomyelinase Activity

Different stress situations increase the expression of secreted sphingomyelinase. Some of the best known are epithelial cells, which are known to be responsive via SMase in irradiation-caused stress [20]. Two cell lines were chosen for the experiments to test the stress response via secreted SMase: Primary human aortic endothelial, HAoEC, cells as model cells for epithelium, and human prostate cancer cells, PC-3, as a tumor model. While it is well-known that irradiation can activate acid sphingomyelinase release, it was chosen for a stress inducer. Various doses of radiation (from 5 to 30 Grays) were used. A secreted enzyme, acid sphingomyelinase, activity was analyzed from the cell media after radiation treatment. HAoEC cells gave higher acid sphingomyelinase response for radiation, but both cell types secrete acid sphingomyelinase in a dose-dependent manner (Figure 2c). From the data, we can conclude that the radiation treatment increases the levels of secreted sphingomyelinase.

### 2.4. Testing Liposomes in Tumor Models

**Figure 3:**
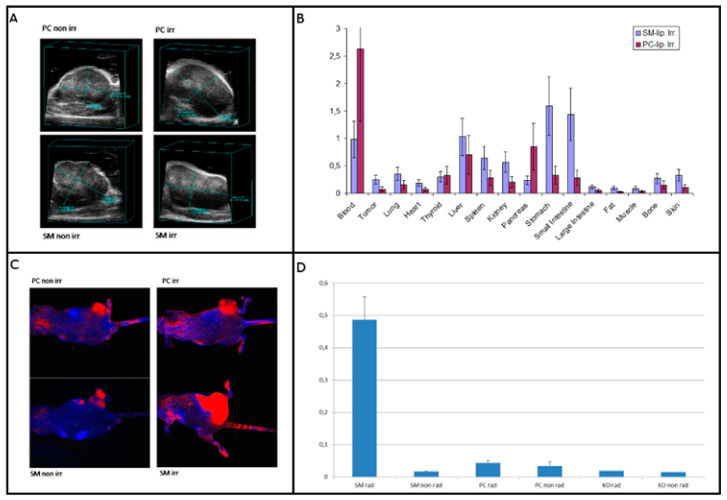
(**A**) presents a morphology from ultrasound analysis of the PC-3 tumors in mice. The upper-left panel represents the tumor treated with control (PC) liposomes without irradiation, and the panel on the upper right shows the tumor treated with both control liposomes and radiation (20Gy). The lower left panel presents a tumor treated with SM-liposomes, and the lower right shows the tumor treated with SM-liposomes and radiation. (**B**) illustrates the biodistribution of SM liposome vs. PC liposome in mice PC-3 tumors two hours after irradiation. Radiolabeled BSA was used as the payload in the liposomes. (**C**) presents a comparison of SM- or PC-liposomes loaded with Alexa Fluor 680 fluorophores. More precisely: Images in the upper row show PC-3 tumors on mice treated with PC-liposomes (left) and with PC-liposomes and radiation (right). In the lower row, tumors are treated with SM-liposomes (left) and SM-liposomes with irradiation (right). The imaging was performed using a Maestro optical camera using spectral un-mixing. (**D**) presents a fluorescent intensity analysis from PC-3 tumor xenograft mice treated with Alexa Fluor 680 labeled liposomes. The area of interest (ROI) was chosen at the tumor site, and as a control, a similar size of ROI was selected at the other hind leg from the same location. Both irradiated and non-irradiated tumors were analyzed similarly. Maximum relative fluorescence intensity RFI was used for analysis.

In addition to release abilities in vitro, we wanted to test the usability in vivo. The biodisribution of the SM-liposomes was studied in athymic nude mice bearing PC-3 prostate xenografts in hind limbs. The animals were randomized by the tumor volume to exclude any size effects. Tumor volumetry was performed with ultrasound. All tumors showed some hypoechogenic areas consistent with necrosis. The tumor volumes varied between 500 and 3500 mm^3^ (Figure 3A). The SM- liposome biodistribution was studied in PC-3 bearing mice 2 h after irradiation, and it was compared to the mice treated with PC -liposome. The irradiation 20 Gy was chosen based on earlier studies [21]. The comparison clearly shows the benefit of using SM-liposomes for tumor-targeting (Figure 3B). Tumor accumulation was more than doubled if compared to PC-liposomes.

### 2.5. Fluorescence Imaging of SM-Liposomes

To analyze which part of the targeting seen in the biodistribution study was due to the enhanced permeability and retention (EPR) effect or the leakage of the vasculature after irradiation, we compared the SM- liposomes to PC- liposomes. In PC-liposomes SM is replaced with PC lipids, so there should not be any active release or targeting function in the tumor treated with them. The irradiation-imaging sequence was performed in a similar way to the biodistribution study. The optical imaging was started 30 min after irradiation, and the liposomes were labeled with an optical marker (Alexa Fluor 680) instead of radiolabeled BSA. The irradiation of the PC-3 tumor-bearing animals was done by X-ray irradiation (20 Gy) of the sedated mice, where only the tumors in the hind legs were exposed to the irradiation. The rest of the body was shielded by lead. Optical imaging with the Maestro was followed after the irradiation of the mice. The fluorescent signals in irradiated tumors were higher in the group that received SM-liposomes compared to the one that received PC liposomes, indicating that the detected extra signals were generated by activation/active targeting of the SM-liposomes (Figure 3C). While we wanted to study the effect of irradiation, we compared the liposomes also in animals that were not irradiated. The irradiated tumors showed considerably brighter signals than the non-irradiated tumors (Figure 3C,D). The spectral analysis and quantification of Alexa 680 in tumors revealed significant differences in the amounts of Alexa 680 in the tumors depending on the radiation of the tumor and a liposome type used as a carrier. Sphingomyelin containing liposomes delivered Alexa 680 significantly better to the irradiated tumors. SM-liposomes function better than control PC-containing liposomes in both cases of tumors (radiated or non-radiated tumors) (Figure 3D).

**Figure 4:**
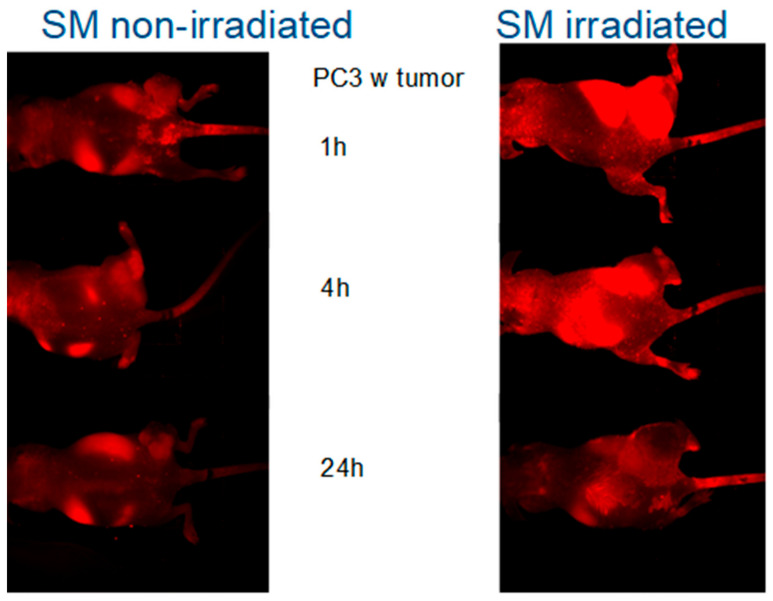
In order to determine whether the radiation impact is time-dependent, the animals were imaged repeatedly 1 h, 4 h, and 24 h after injection of the liposomes. One tumor got irradiation (upper leg in images) while the other tumor site was protected from irradiation with lead (lower legs). The left panel presents mice bearing PC-3 tumor in both legs and treated with SM-liposomes images were taken 1 h, 4 h, and 24 h (images from up to down, respectively) after the treatment. The right panel presents similar animals but treated in addition to SM-liposomes with 20 Gy radiation. The images were taken 1 h, 4 h, and 24 h (images from up to down, respectively) after the treatments.

To further investigate the possibility of using the SM-containing liposomes for detection of cell damage in vivo, we used nude mice bearing subcutaneous PC-3 tumors: Each animal had two tumors, one on each side of the hind leg. One of the tumors was irradiated with 20 Grays, while the other one was protected from irradiation by lead shielding. Mice were injected with Alexa 680 containing liposomes prior to irradiation with 20 Grays followed by imaging. There was a significant homing of the Alexa Fluor 680 fluorophore to the tumors (Figure 4) after irradiation. In order to determine whether the SMase impact was time-dependent, the animals were imaged repeatedly 1 h, 4 h, and 24 h after injection. The liposomes were given prior to 20 Gy irradiation, and the irradiation was focused only on the one tumor site. The data showed the highest signals at the early time points and a steady clearance in all of the cases after several hours but giving higher fluorescence in the irradiated animal group than the non-irradiated group (Figure 4). The highest effect in early time points fits well to the previous cell data, which show that aSMase activation is an early step in a process [12,13,14]. Sphingomyelin-containing liposomes can be used to track tissue damages, and the targeting efficacy is higher in SM-liposomes if compared to the control (PC) liposomes in living animals.

## 3. Discussion

We wanted to test our SM-liposome both in vivo and in vitro to study the proof of concept for (1) the liposomal release and (2) disease targeting using fluorescence methodology. Liposomes can be used as carriers for fluorescent markers like described here or as a drug carrier in the future. Liposome also offers a platform for multimodality systems where for example, imaging and drug delivery can be combined. It has been shown that apoptotic and stressed cells transport SMase in the outer leaflet of the apoptotic cells [13,14], a condition that does occur in healthy cells. Because apoptosis is a process that occurs in the tumors spontaneously and is increased after chemotherapy or radiotherapy, it would be helpful to know the rate of cell stress and apoptosis after a treatment to have a measure for therapeutic efficacy.

The proposed mechanism of action is that the SM in the lipid layer will be cleaved to ceramide upon contact with SMase. Ceramide has been reported to form ceramide-enriched microdomains in model membranes [6,8,9]. Ceramide also makes a membrane leakier [5]. The SMase/ceramide-related aggregations are not shown only in model membranes but also in cells [13,14,22,23,24]. Lately, there have been new methods to target and detect ASMase levels from tissues [25,26].

Our study showed that PC-3 cells secrete SMase in a dose-dependent manner when irradiated from 5 to 30 Gy. We also observed that HAoEK endothelial cells secrete SMase in a similar manner but more when irradiated. Therefore, both endothelial cells and the cancer cells could be the source of the SMase during irradiation-induced stress. Fluorescence measurements in in vitro studies where SMase-treated liposomes were compared to non-SMase treated liposomes show increased fluorescence of roughly two-fold. This is because the release of Cy 5.5, which is auto-quenched inside liposomes, increases the fluorescence when released and being free from quenching. The heat-inactivated SMase did not raise the fluorescence as expected.

Free Alexa 680 was rapidly cleared from the circulation in all time points used in this study and could be detected only in the bladder. In the case of liposomal formulation, clear homing of the liposomal Alexa 680 to the tumors could be seen. This was possibly due to the passive EPR effect. However, there was a clear tendency of increased targeting of the fluorophore to the irradiated tumors in the case of SM-containing liposomes. This phenomenon could not have been seen with control PC-liposomes even when tumors were irradiated. This suggests that there are SM-specific mechanisms for the delivery of the payload to the tumors. When irradiated, the two possible sources for SMase secretion are the endothelial vessels in the tumor area and the tumor cells themselves.

Liposomes, micelles, and other nanoparticles can be used as carriers for imaging tracers. The results from imaging can further be used for the development and optimization of the delivery systems for therapy. This can lead to a more beneficial pharmacodynamics profile of the drugs, DNA/RNA, or potent peptides if compared to free drugs or liposomal delivery systems alone. The SMase-triggered liposome disruption could allow the drug to be administered before radiation, and the drug would be released only on the site of the radiation immediately when the radiation occurs. In a theragnostic approach, the imaging component can be used to first evaluate the drug delivery capabilities followed by treatment approaches. Improving drug delivery for better therapeutic outcomes is an eminent goal. However, the choice of encapsulated drugs is an issue by itself. More and more valuable RNA-based compounds are produced by the scientific community and a cell stress responsive liposome could be a viable choice of vehicle for them. On the other hand, RNA encapsulation is a task by itself, possibly needing changes in the surface charge, and the RNA has to be chosen wisely. There are also classical Doxorubicin liposomes that could be used as a benchmark, but in the encapsulation process of the doxorubicin they are crystallized and not immediately fully soluble after release. In addition, the immunological situation in the target tissue is one parameter that has to be evaluated. The liposomal delivery system affects the immunological activity of the disease site, making the choice of the encapsulated drug even more important. In the future, this SMase-activated release could be projected to be used in multiple theranostic and therapeutic delivery products in clinic settings.

## 4. Materials and Methods 

### 4.1. Liposomal Preparation

Lipids were purchased from Avanti Polar Lipids (Alabaster, AL, USA) and bacterial SMase produced by *B. cereus* from Sigma Aldrich (Germany). Liposomes were made from a lipid mixture consisting of 20 μMoles of total lipids (4/3/3; DSPC/Cholesterol/SM in mol/mol ratios). Liposomes were prepared as described earlier [15]. Shortly, lipids stored in chloroform were pipetted to a round-bottomed flask, dried under nitrogen, and lyophilized for 24 h to remove trace amounts of chloroform. Lipids were hydrated in PBS solution for 30 min in 60 C water bath. The buffer contained Cy 5.5, hairpin DNA, Alexa 680 streptavidin, or radiolabeled albumin, depending on the conducted experiment. Extrusion was performed 11 times through 100 nm polycarbon membrane by using a small volume extruder. Formed liposomes were purified from the unbound compounds with a PD 10 Sephadex column (GE Healthcare, Braunschweig, Germany). The 1.5 mL fractions were collected. Liposome size measurements were done using dynamic light scatter (Malvern, Worcestershire, UK), and it revealed an average hydrodynamic diameter of 100–200 nm for the liposomes.

The release studies were based on the self-quenching of the fluorophores loaded to the lumen of the liposomes. The liposomes containing Cy 5.5 were incubated with different amounts of SMase (1 U, 0.5 U, and 0.1 U) and control samples with heat-inactivated SMase. The fluorescence was measured with Tecan Saphire plate reader (Tecan Group, Männedorf, Switzerland) after 30 min treatment. In order to obtain a good correlation between SMase activity and signal intensity, different marker systems were used: DNA beacon containing a hairpin loop with a CY7-Dabcyl quenching pair was also used as the payload for liposomes.

SMase activity was visualized by a fluorometric acid SMase detection kit (Cayman Chemicals, Ann Arbor, MI, USA) according to the instructions provided. The kit utilizes coupled enzymatic reactions, which produce a fluorescent product monitoring the sample’s sphingomyelinase activity. Released SMase activity was measured from the cell media of the irritated primary human aortic endothelial cells (HAoEC) or PC-3 cells. The different doses ranging from 0 to 30 Gy were used, the fluorescent signal intensity correlating with SMase enzyme activity was measured with a Tecan Saphire plate reader 10 min after irradiation.

### 4.2. Mouse Tumor Model

All animal experiments were conducted in compliance with Institutional Animal Care and Use Committee (IACUC) guidelines. And by the Memorial Sloan Kettering Cancer Center NY;NY Institutional animal care and use committee protocol from 2020 number 8602020, and followed National Institutes of Health guidelines for animal welfare. Male athymic nu/nu mice (NCRNU-M, 20–22 g, 6–8 weeks old) were obtained from Taconic Farms Inc. (Hudson, NY, USA) and were allowed to acclimatize at the MSKCC vivarium for one week before the tumor implantation. Athymic nude mice bearing PC-3 prostate xenografts were used for in vivo evaluation of SM liposomes. PC-3 is an established grade 4 human prostatic adenocarcinoma. 400,000 cells in 200 µL matrigel were injected subcutaneously into both hind limbs. Experiments were performed three weeks after injection.

The mice were randomized based on the tumor volume to exclude the tumor size effects. For this purpose, tumor volumetry was performed with a Vevo 770 high-frequency ultrasound device (Fujifilm, Toronto, ON, Canada).

### 4.3. Tumor Irradiation and SM-Liposome Biodistribution

The sedated mice were irradiated with an X-ray with an administered dose of 20 Gy. Only the tumors and the hind legs were exposed to the irradiation. The rest of the body was shielded by lead.

The liposomal biodistribution study was performed 2 h after irradiation. The SM-liposomes loaded with radiolabeled BSA [16] were used. As a control, we had the same liposomes, only lacking the SM, which was replaced with PC-lipid. Radiolabeled BSA was used as a payload in both liposomes. The animals bearing PC-3 tumors were irradiated with 20 Gy. Immediately after irradiation, radiolabeled liposomes were administered via tail vein injection to the mice, and after two hours, the animals were sacrificed, and organs were collected for radiological analysis by a gamma counter.

### 4.4. Fluorescence Imaging

A comparison of SM- and PC-liposomes in tumor targeting was proceeded with the liposomes loaded with Alexa Fluor 680 fluorophores. Optical imaging followed the irradiation (20 Gy) of the PC-3 tumor-bearing mice. The liposomes were administered immediately after radiation by tail vein injection. The mice were imaged with a Maestro fluorescence camera. Image analysis was performed according to Maestro software. The area of interest (ROI) was chosen at the tumor site, and as a control, a similar size of ROI was selected at the other hind leg from the same location. Both irradiated and non-irradiated tumors were analyzed similarly. Maximum relative fluorescence intensity RFI of the site was used.

To further investigate the possibility of using the SM-containing liposomes to detect cell damage in vivo, we used nude mice bearing subcutaneous PC-3 tumors. Each animal had two tumors, one on each side of the hind leg. Mice were injected with Alexa Fluor 680 fluorophore-containing SM-liposomes prior to irradiation with 20 Grays. Only one tumor leg was irradiated. Other parts of the body were shield with lead. The tumor on the opposite leg is used as a control for the irradiation effect. Images were taken immediately after irradiation, i.e., 30 min after injection. Imaging was performed using a Maestro optical camera and using spectral un-mixing. In order to determine whether the radiation impact was time-dependent, the animals were imaged repeatedly 1 h, 4 h, and 24 h after injection of the liposomes and irradiation of one tumor site according to the described protocol.

## 5. Patents

EP2948132B1.

## Data Availability

All the raw data is available upon request.

## Abbreviations

SM	Sphingomyelin
PC	phosphatidyl choline
GUV	giant unilamellar vesicle
EPR	enhanced permeability and retention
SMase	Sphingomyelinase
PET	positron emission tomography
MRI	magnetic resonance imaging
DLS	dynamic light scattering
HAoEC	human aortic endothelial cells
PC-3	Caucasian prostate adenocarcinoma
